# All-in-one smart dressing for simultaneous angiogenesis and neural regeneration

**DOI:** 10.1186/s12951-023-01787-5

**Published:** 2023-02-03

**Authors:** Tiejun Yuan, Minhong Tan, Yang Xu, Qiyao Xiao, Hui Wang, Chen Wu, Fulun Li, Lihua Peng

**Affiliations:** 1 College of Pharmaceutical Sciences, Zhejiang University, Hangzhou, 310058 People’s Republic of China; 2 State Key Laboratory of Quality Research in Chinese Medicine, Macau University of Science and Technology, Macau, People’s Republic of China; 3 College of Materials Science and Engineering, State Key Laboratory of Silicon Materials, Zhejiang University, Hangzhou, 310027 People’s Republic of China; 4 Department of Dermatology, Yueyang Hospital of Integrated Traditional Chinese and Western Medicine, Shanghai University of Traditional Chinese Medicine, Shanghai, 200437 People’s Republic of China

**Keywords:** All-in-one smart dressing, Multi-functional niche, Angiogenesis, Neural regeneration

## Abstract

Wound repair, along with skin appendage regeneration, is challenged by insufficient angiogenesis and neural regeneration. Therefore, promoting both proangiogenic and neuro-regenerative therapeutic effects is essential for effective wound repair. However, most therapeutic systems apply these strategies separately or ineffectively. This study investigates the performance of an all-in-one smart dressing (ASD) that integrates angiogenic functional materials and multiple biological factors within a light crosslinked hydrogel, forming a multi-functional dressing capable of facilitating simultaneous micro-vascularization and neural regeneration. The ASD uses a zeolite-imidazolate framework 67 with anchored vanadium oxide (VO_2_@ZIF-67) that allows for the on-demand release of Co^2+^ with fluctuations in pH at the wound site to stimulate angiogenesis. It can simultaneously release CXCL12, ligustroflavone, and ginsenoside Rg1 in a sustained manner to enhance the recruitment of endogenous mesenchymal stem cells, inhibit senescence, and induce neural differentiation to achieve in situ nerve regeneration. The ASD can stimulate rapid angiogenesis and nerve regeneration within 17 days through multiple angiogenic and neuro-regenerative cues within one dressing. This study provides a proof-of-concept for integrating functional nanomaterials and multiple complementary drugs within a smart dressing for simultaneous angiogenesis and neural regeneration.

**Supplementary Information:**

The online version contains supplementary material available at 10.1186/s12951-023-01787-5.

## Introduction

Skin contains a dense network of blood vessels and nerve fibers that are integral to maintaining systemic homeostasis. Various skin injuries, such as wounds, burns, diabetic ulcers, and phlebitis of the lower extremities, can compromise vascular and neural systems, causing limited regeneration of skin functions related to the somatosensory system [1, 2]. Inhibited angiogenesis also retards the transport of oxygen and nutrients required for skin repair [3, 4], leading to slow or even non-healing wounds. Therefore, effective wound repair requires both angiogenic and neuro-regenerative strategies in skin appendage regeneration. Most therapeutic systems apply growth factors, such as vascular endothelial growth factor (VEGF) [5] or neural growth factor (NGF) [6], separately and inaccurately, leading to limited therapeutic efficiency with drug resistance, host immune rejection, and, sometimes, carcinogenesis. Therefore, an intelligent system that effectively promotes angiogenesis and neural regeneration in an on-demand and controlled manner would be highly desirable. Moreover, considering the cancer risk and side effects associated with growth factors or synthetic chemical compounds, naturally sourced bioactive materials and compounds are expected to be safer alternatives to conventional therapeutics.

Cobalt ions (Co^2+^) have been shown to stabilize hypoxia-inducible factor-alpha (HIF-1α), thereby promoting the expression of VEGF and stimulating angiogenesis [7–9]. Zeolitic imidazolate framework-67 (ZIF-67) [10] can actively release Co^2+^ in acidic microenvironments, making it a promising material to induce targeted angiogenesis, especially in the early stages of wounding [11, 12], and provide a safer alternative to conventional angiogenic growth factors [13]. Based on these properties, In this study, for the first time, we designed a hollow microsphere vanadium dioxide (VO_2_) [14] anchored with nano-scaled ZIF-67 (VZ) as a smart angiogenic inducer, to promote angiogenesis by releasing Co^2+^ in response to the acid microenvironment in wound microenvironment.

Current strategies to stimulate nerve regeneration, such as autologous nerve grafting [15], tissue-engineered skin transplantation [16], porous scaffold implantation, and electrical stimulation, are limited either by their low efficacy, high cost, and/or safety risks. In contrast, in situ tissue regeneration has the advantages of recruiting endogenous stem cells to the wound site for directed differentiation and regeneration [17]. Bone marrow-derived mesenchymal stem cells (BMSCs) are important seed cells for in situ tissue regeneration because of their multi-directional differentiation potential. Nearly all tissues have a self-renewing multipotent stem cell population that is maintained in a specialized microenvironment, or niche [17]. These stem cell niches can be disrupted by disease, acute injury, burns, inflammation, and ulcers, among others. For effective wound repair, it is essential to establish a microenvironment that maintains the stem cell pool and directs stem cell behavior around the target. Chemokine C-X-C motif ligand 12 (CXCL12) is an important active protein that recruits BMSCs by specifically binding to the chemokine receptor CXCR4 [18–21]. However, the accumulated number of endogenous BMSCs in circulation is limited and affected by senescence and proliferation [22, 23], which significantly decreases their differentiation potential. Therefore, the addition of not only therapeutics to recruit BMSCs but also retard senescence and promote differentiation is crucial for the success of in situ regeneration strategies.

Growth factors and gene modifications are the main strategies utilized to promote proliferation or retard senescence in stem cells, but their use is constrained by high cost, short duration of effect, tumorigenic risk, and/or complicated methods of manipulation [24, 25]. Medicinal plants have recently been shown to effectively regulate division and senescence in stem cells. Medicinal plant compounds have attracted increasing attention owing to their low cost, high safety, and remarkable therapeutic activities [26, 27]. In previous studies, including our own, ligustroflavone (Lig) and ginsenoside Rg1 (Rg1) were shown to inhibit senescence, promote proliferation, and induce neural differentiation in stem cells [28, 29].

Hydrogels are known for their unique properties, such as high sensitivity to physiological environments, hydrophilic nature, soft tissue-like water content, adequate flexibility, and responsiveness to environmental stimuli, such as temperature, pH, and ionic strength [30]. Moreover, owing to the moist environment of the wound and porous structure of the hydrogel, bioactive substances can be released in a controlled manner to construct a biomimetic niche that regulates the stem cell population [31].

**Fig. 1:**
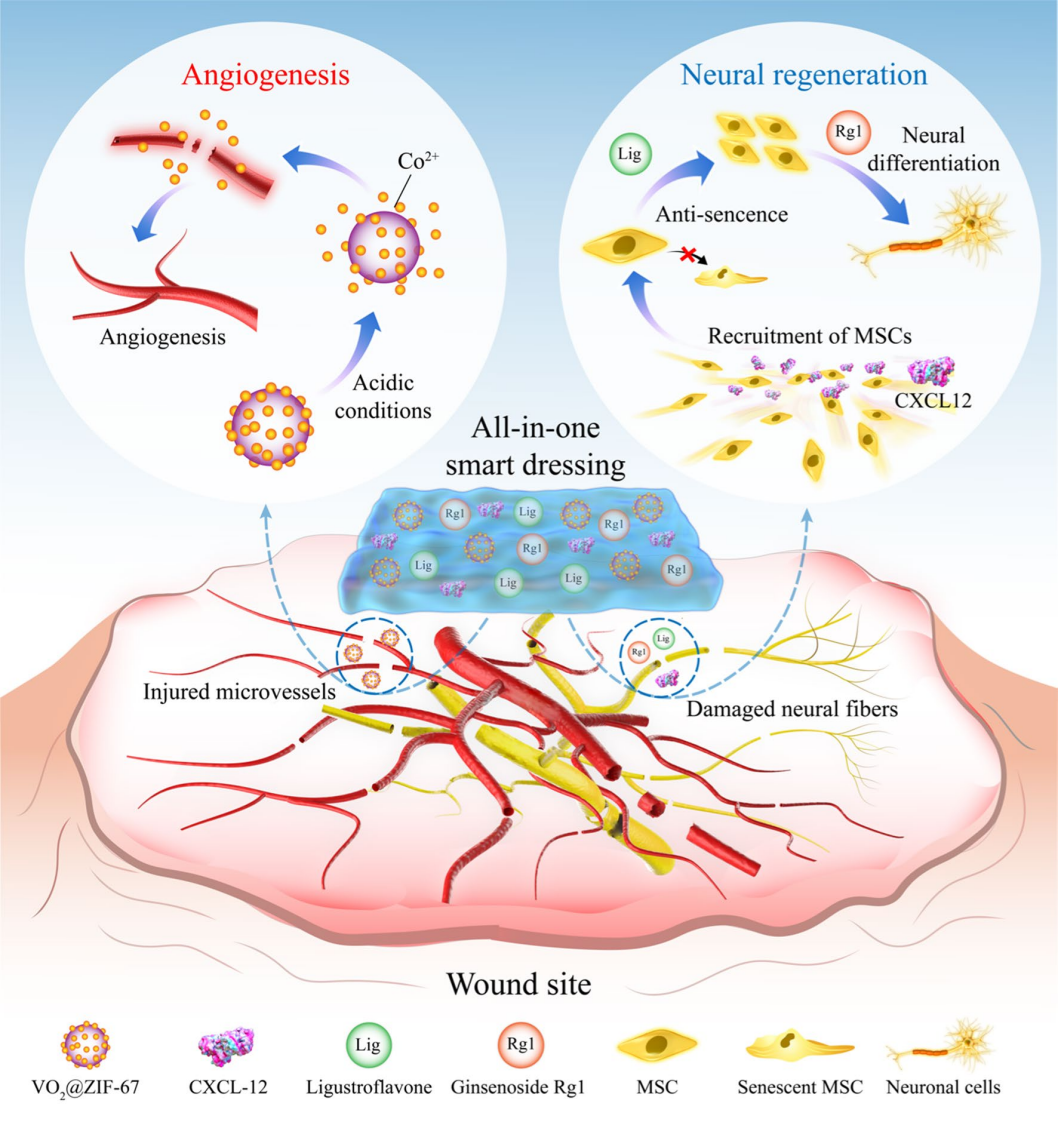
Schematic illustration of the responsive release of Co^2+^ and controlled delivery of CXCL12, Lig, and Rg1 from the ASD, which enhance angiogenesis and neural regeneration at the injured site

In this study, we propose an all-in-one smart dressing (ASD) that incorporates VZ, Lig, Rg1, and CXCL12 within a light-controlled in situ crosslinked hydrogel, gelatin methacryloyl (GelMA), which has physicochemical properties similar to those of the extracellular matrix (Fig. 1). On the one hand, the ASD effectively recruited stem cells to the wound site and regulated their behavior. The ASD presents multiple neural regenerative cues along with angiogenic signals in one smart dressing that comfortably fits irregular wound and ulcer configurations, providing a proof-of-concept for an artificial niche to stimulate both angiogenesis and neural regeneration, with great potential for application in regenerative medicine.

## Results

### Construction and characterization of VO_2_@ZIF-67

**Fig. 2:**
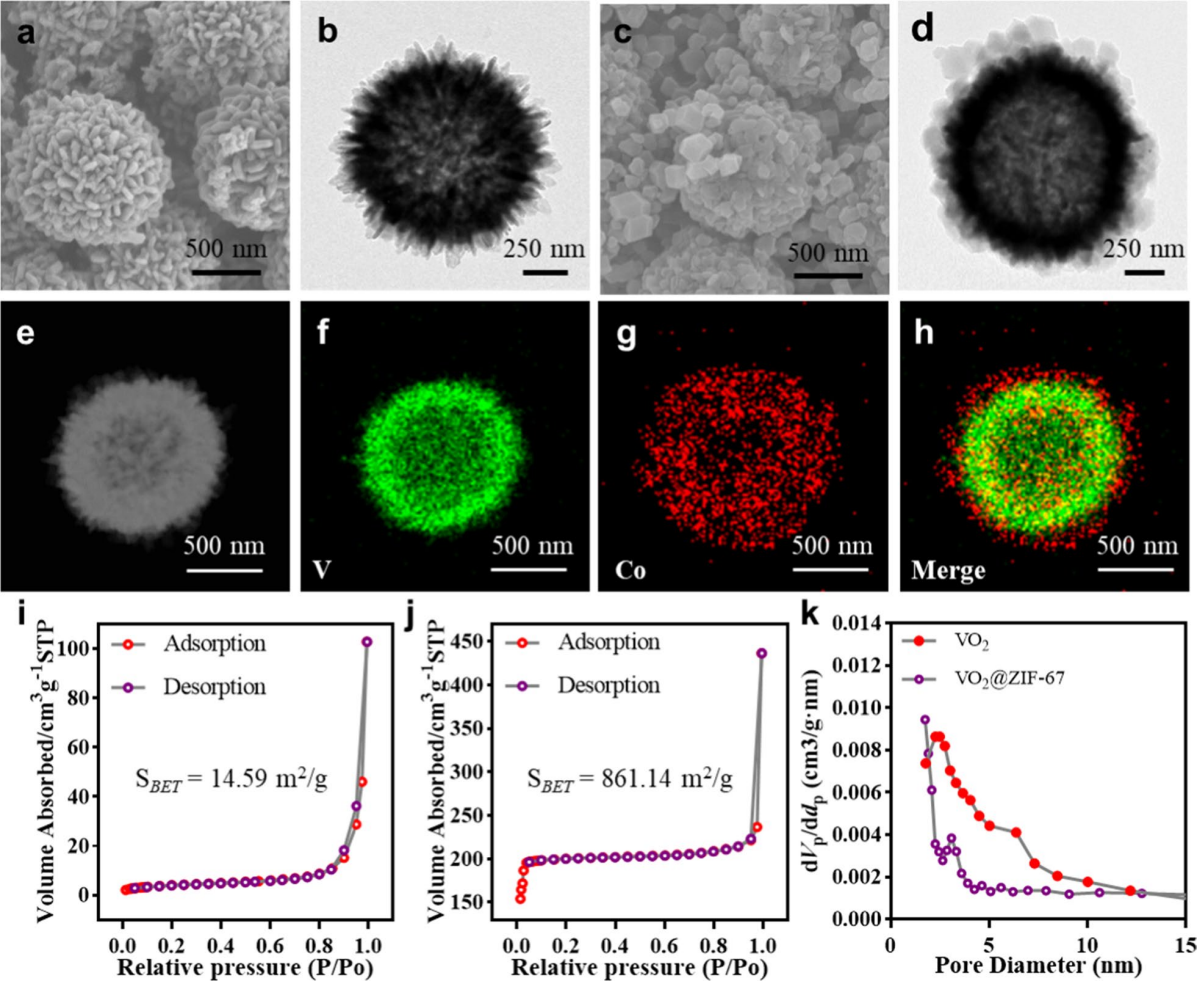
Structural characterization of the VZ. **a** SEM and **b** TEM images of VO_2_ particles. **c** SEM and **d** TEM images of VZ particles. **e** High-angle annular dark field (HAADF) and **f**–**h** energy dispersive spectroscopy (EDS) images of VZ particles. N_2_ absorption–desorption isotherms of **i** VO_2_ and **j** VZ particles. **k** Pore diameter distributions of the VZ

VO_2_@ZIF-67 (VZ) was prepared by in situ growth of ZIF-67 on VO_2_ surfaces, forming a hierarchical nano/micro composite. The VO_2_ particles were spherical and hollow (Fig. 2a) with a size of 953.6 nm (Additional file 1: Fig. S1). The prickles distributed on the VO_2_ surfaces (Fig. 2a, b) provided sites for the nucleation and growth of ZIF-67, which formed a dodecahedral shell-like structure tightly attached to the hollowed VO_2_ core (Fig. 2c, d). The composite particle had a size of approximately 17 µm (Additional file 1: Fig. S1). High-angle annular dark field imaging (Fig. 2e) and elemental mapping further demonstrated the hierarchical structure of the VZ, of which V atoms were concentrated at the edges (Fig. 2f), while Co had an even and greater distribution than V (Fig. 2g, h). The hollow structure of VO_2_, porous nature of ZIF-67, and hierarchical arrangement collectively contributed to the increased surface area (Fig. 2i, j) and porosity (Fig. 2k) of the VZ. This maximizes the surface area for interaction with the surrounding microenvironment, resulting in enhanced bioactivity. These results indicate that we successfully constructed the micro/nano composite VZ.

### Construction and characterization of the ASD

**Fig. 3:**
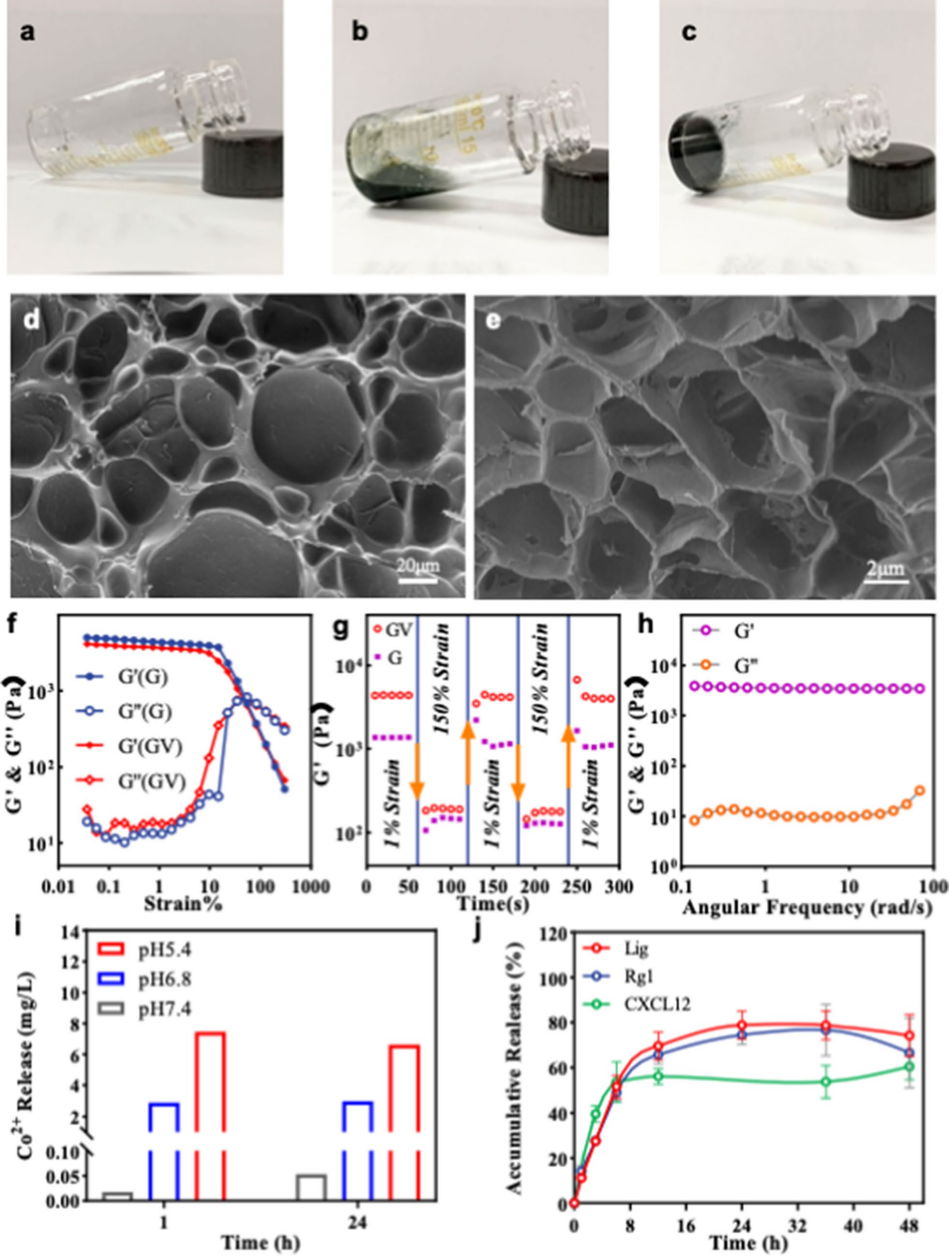
Characterization of the ASD. **a** Liquid GelMA pre-gel. **b** Pre-gel suspension of GelMA and drug. **c** ASD after cross-linking. **d** SEM image of the surface. **e** SEM image of the internal structure of the GelMA hydrogel. **f** Strain amplitude sweep test (γ = 0.1–300%) at a fixed angular frequency (1 rad/s) of the Gel (G) and Gel/VZ (GV). **g** Alternate step strain sweep test with little strain (γ = 1.0%) to greater strain (γ = 150%) over 60 s for every strain interval at a fixed angular frequency (1 rad/s). **h** Rheological performance of the Gel/VZ. **i** Cumulative release of Co^2+^ from the Gel/VZ at pH 5.4, pH 6.8, and pH 7.4. **j** CXCL12, Lig, and Rg1 release behavior from the ASD

Hydrogels provide a favorable environment for wound healing and serve as drug reservoirs for tissue regeneration [32]. We used a GelMA [33] hydrogel as a scaffold, which consists of gelatin (the hydrolyzed product of collagen) that retains functional amino acids, such as glycine-aspartic acid and matrix metalloproteinase-responsive peptides, which provide sites for cell adhesion and enzyme degradation in vivo [34–38]. In addition, the methacryloyl functional groups endow GelMA prepolymer solutions with free-radical-induced chemical crosslinking [33]. Liquid GelMA pre-gel (Fig. 3a) forms suspension with the drug (Fig. 3b) and then crosslinks to form ASD (Fig. 3c). Meanwhile, the GelMA scaffold showed reticular porous structures (Fig. 3d, e) that could effectively load functional compounds and bioactive drugs, while retaining water.

To optimize the lifespan of the dressing, the hydrogel scaffold must bear certain external mechanical forces. The Gel/VZ could withstand greater external mechanical force than the gel scaffold alone (Fig. 3f). The storage modulus (G′) of the Gel/VZ rapidly decreased from 4368.4 to 184.0 Pa at 150% high strain and was rapidly restored to the initial level after the strain decreased to 1% (Fig. 3g), indicating that the original form of the Gel/VZ is rapidly restored following strain. Moreover, the G′ of the Gel/VZ in the range of ω = 0.1 − 100 rad/s was greater than the loss modulus (G″) (Fig. 3h), indicating the stiff mechanical property of the Gel/VZ. In 24 h, the Gel/VZ released 0.05 mg/L Co^2+^ at pH 7.4, 3.0 mg/L at pH 6.8, and 7.5 mg/L at pH 5.4 (Fig. 3i), indicating the effective pH-triggered release of Co^2+^ release from the Gel/VZ.

We loaded the Gel/VZ with CXCL12, Lig, and Rg1 to generate the ASD (Gel/VZ-CLR). The Gel/VZ had a sustained release of CXCL12, Lig, and Rgl (Fig. 3j). Over 48 h, the Gel/VZ continuously released up to 60.48 ± 5.54% CXCL12. Over 36 h, the Gel/VZ continuously released up to 78.72 ± 6.38% and 76.67 ± 11.44% of Lig and Rgl, respectively. These results indicate that the porous structure of the VZ dispersed across the gel scaffold forms an efficient reservoir for the controlled delivery of multi-therapeutics with different physicochemical features.

### Angiogenic effects of the ASD

**Fig. 4:**
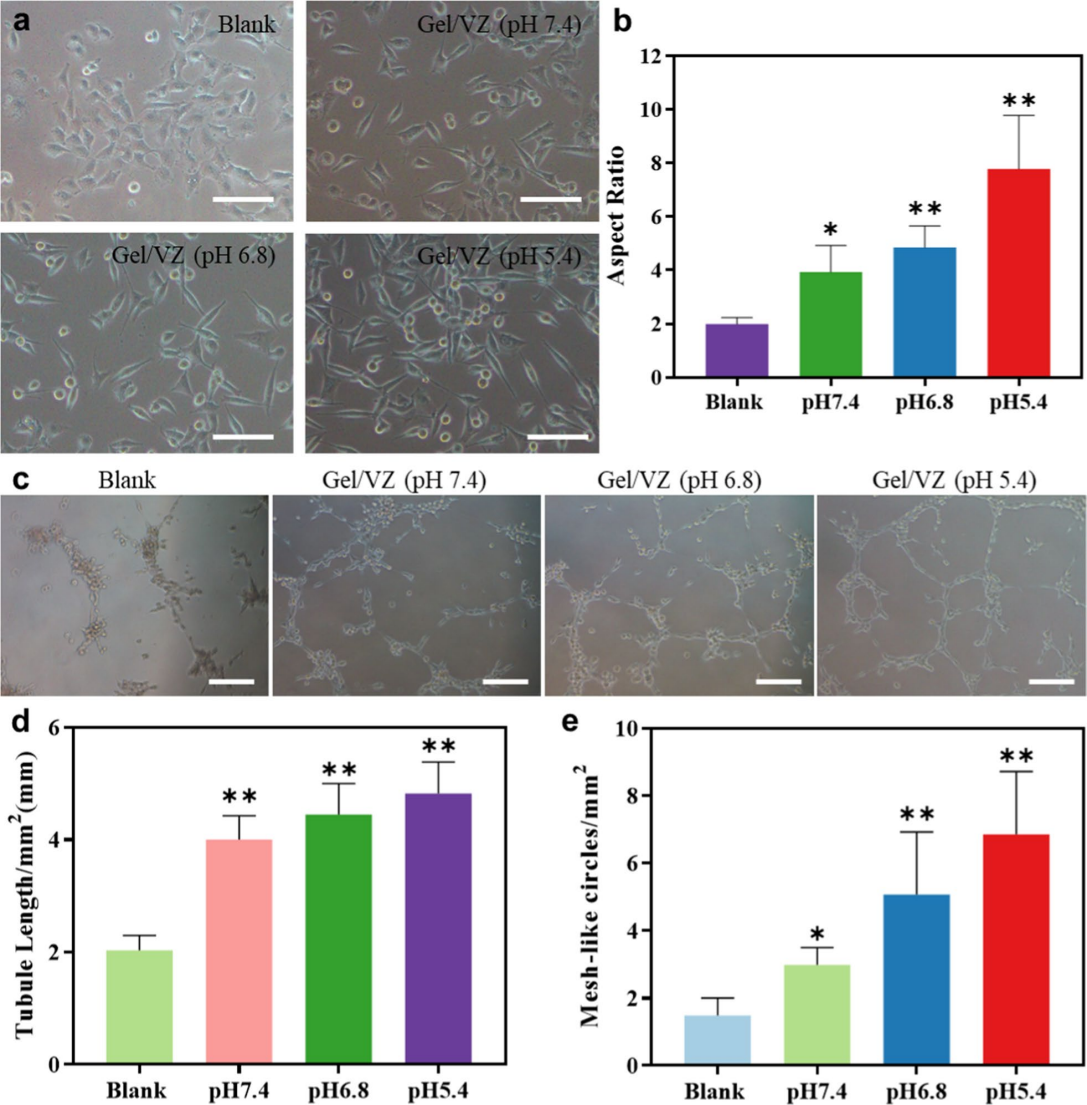
Angiogenic effects of the Gel/VZ. **a** Light microscopy images (Scale bar = 100 μm), **b** cellular aspect ratio, **c** light microscopy images of HUVECs during tubule formation (Scale bar = 200 μm), **d** semi-quantitative tubule length, and **e** mesh-like circles of HUVECs after treatment with PBS or Gel/VZ at pH 7.4, 6.8, or 5.4. *p < 0.05, **p < 0.01, ***p < 0.001 compared to the Blank group

Human umbilical vein endothelial cells (HUVECs) incubated with the Gel/VZ exhibited different degrees of morphological changes (Fig. 4a). Compared with a blank group, Gel/VZ significantly increased the cell aspect ratio by 1.98-, 2.44-, and 3.91-times at pH 7.4, 6.8, and 5.4, respectively (Fig. 4b), which is a manifestation of cell polarization and corresponds to the tubule formation potential of the cells [39, 40]. Compared with phosphate-buffered saline (PBS)-treated HUVECs, the Gel/VZ significantly increased the tubular length by 2.0-, 2.2-, and 2.4-times at pH 7.4, 6.8, and 5.4 (Fig. 4c, d), and the number of reticular tubules by 2.0-, 3.4-, and 4.6-times, respectively (Fig. 4e). These results demonstrate that we successfully constructed a functional Gel/VZ with a pH-responsive release of Co^2+^.

### ASD promotes recruitment, proliferation, and neural differentiation of BMSCs in vitro

**Fig. 5:**
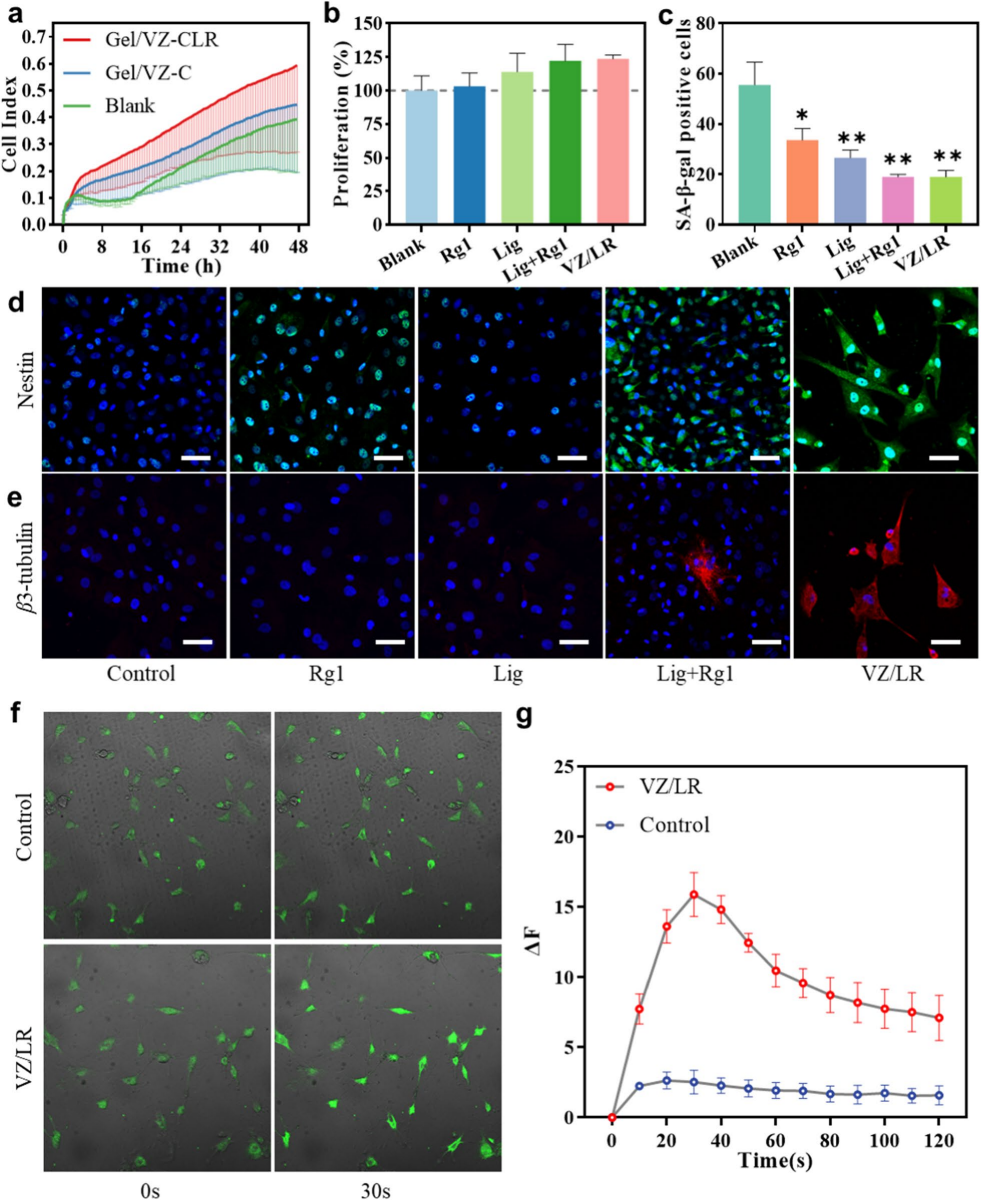
Behavior of rBMSCs according to each treatment. **a** rBMSC migration. **b** rBMSC proliferation. **c** Quantitative analysis of SA-β-gal-positive cells. **d** Nestin in 7 days and **e** *β*3-tubulin in 14 days immunofluorescence (Scale bar = 50 μm). **f** Ca^2+^ expression. **g** Ca^2+^ oscillation in cells under a high concentration of KCl stimulation. *p < 0.05, **p < 0.01, ***p < 0.001 compared to the Blank group

We compared the chemotactic effects of the Gel/VZ loaded with CXCL12 (Gel/VZ-C) and Gel/VZ loaded with CXCL12, Lig, and Rg1 (Gel/VZ-CLR) on rat-derived BMSCs (rBMSCs). The migration of rBMSCs in the blank control group gradually stabilized over 0–16 h (Fig. 5a), while Gel/VZ-C and Gel/VZ-CLR effectively enhanced the migration of rBMSCs, with the cell index of the Gel/VZ-C and Gel/VZ-CLR groups enhanced by 1.1- and 1.8-fold, respectively, which might be attributed to the sustained release of CXCL12 by the Gel/VZ-C (Fig. 3h). Local stem cell pools are essential in stem cell-based regenerative medicine and their maintenance requires strict regulation of cell proliferation and senescence [41]. Compared to the blank group, the proliferation of stem cells was enhanced in all of the tested groups, including those treated with Lig alone, Rg1, a combination of Lig and Rg1 (LR), and a combination of VZ, Lig, and Rg1 (VZ-LR) (Fig. 5b). Meanwhile, the number of SA-β-gal-positive cells was significantly reduced upon treatment with Rg1, Lig, LR, and VZ-LR (Fig. 5c and Additional file 1: Fig. S2).

Based on the result that CXCL12, Lig, and Rg1 promote the replenishment of stem cell pools, we investigated the influence of Lig, Rg1, and VZ treatment on the neural differentiation of BMSCs. We did not detect nestin in BMSCs treated with PBS or Lig, indicating that BMSCs could not differentiate into neural precursors/stem cells without the induction of neural differentiation signals (Fig. 5d). In contrast, nestin expression was detected in BMSCs after 7 days of treatment with Rg1, indicating that Rg1 can induce stem cells to differentiate into neuron-like cells. Moreover, nestin expression was enhanced by stimulation with LR or VZ-LR. This enhanced neural differentiation of BMSCs may be attributed to the anti-senescence effect of Lig, which maintains the multipotency of BMSCs by inhibiting replicative senescence, thus providing a synergistic effect in the neural differentiation of BMSCs induced by Rg1. *β*3-Tubulin—the marker for mature neural cells [42, 43]—expression was not detected in Lig- or Rg1-stimulated stem cells after 14 days (Fig. 5e). In contrast, the expression of *β*3-tubulin was detected in the LR or VZ-LR groups. The expression of nestin and *β*3-tubulin in BMSCs stimulated by VZ-LR was much greater than that stimulated by LR alone, which may be attributed to the enhanced bioavailability of Lig and Rg1 in the VZ. In contrast to untreated cells, BMSCs treated with VZ-LR exhibited obvious electrophysiological characteristics related to Ca^2+^ influx (Fig. 5f, Additional file 2: Movie S1 and Additional file 3: Movie S2), the ∆F of which was 6.28-fold higher than that of untreated BMSCs (Fig. 5g). The above results suggest that Rg1-induced neural differentiation in rBMSCs can be enhanced by Lig, thereby acting synergistically in retarding senescence and promoting neural differentiation. These results suggest that the combination of Rg1, Lig, and VZ can effectively induce the neural differentiation of stem cells.

### ASD promotes the recruitment and proliferation of BMSCs in vivo

**Fig. 6:**
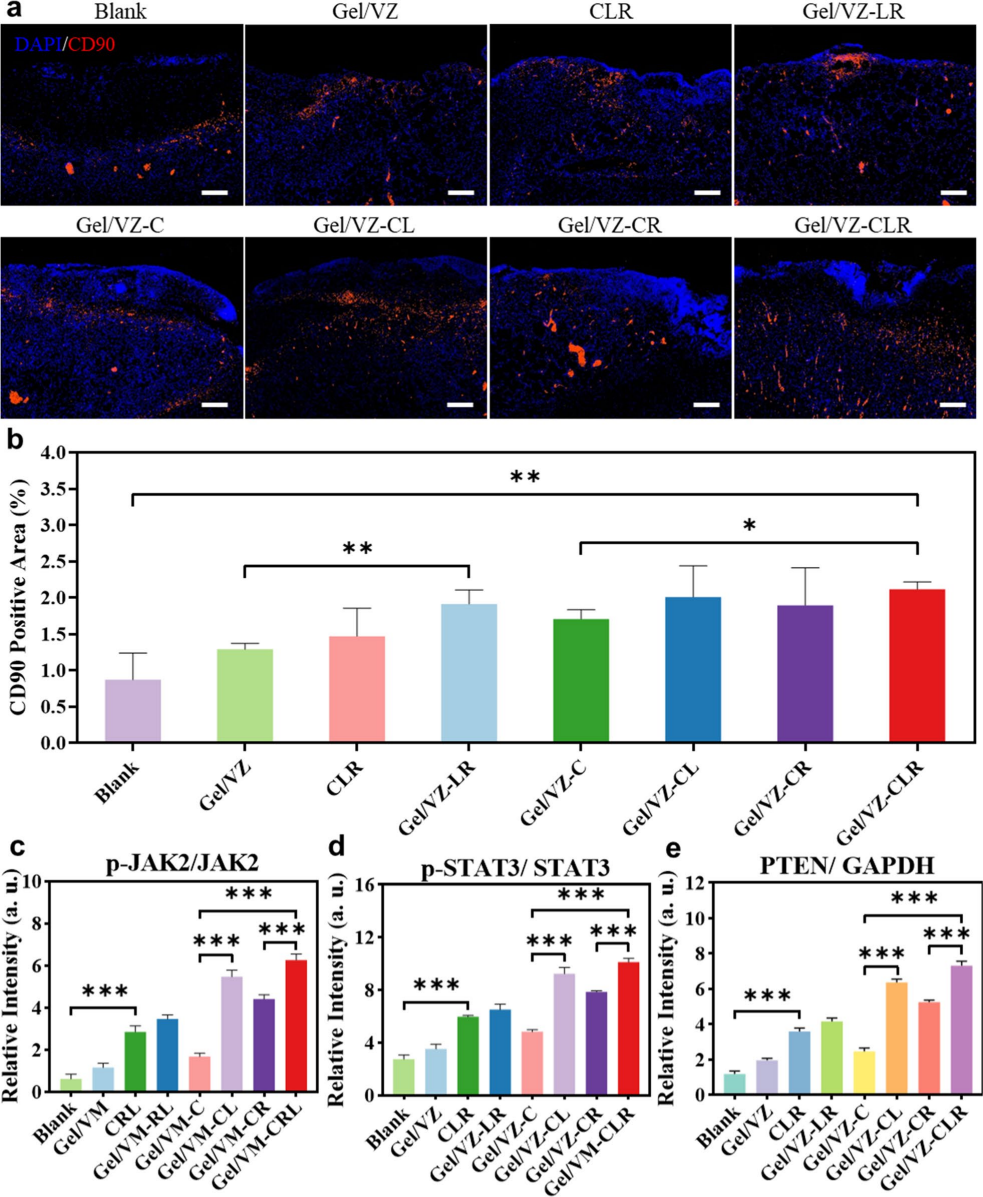
In vivo recruitment and proliferation of BMSCs in wound tissue. **a** Immunofluorescence staining and **b** semi-quantitative fluorescence-positive area of CD90 (red) on day 3 (Scale bar = 200 μm). Relative expression levels of **c** p-JAK2, **d** p-STAT3, and **e** PTEN on day 3. *p < 0.05, **p < 0.01, ***p < 0.001 compared to the Blank group

The recruitment of stem cells at wound sites plays a vital role in in situ skin regenerative strategies. To verify the in vivo influence of ASD on the recruitment and proliferation of BMSCs, we collected skin tissue from wound sites on the third day post-treatment with ASD and detected CD90—a classical marker of BMSCs—to examine the migration of BMSCs [44, 45]. The CD90 signals in the Gel/VZ-CLR group had a wider distribution and greater overall area compared to the blank group (Fig. 6a, b). Only a small number of CD90^+^ cells were observed in the wound sites of the Blank and Gel/VZ groups, which may reflect that only very few BMSCs migrated to the wound sites in the absence of proper chemotactic signals. More CD90^+^ cells were observed in the Gel/VZ-LR group than in the Gel/VZ group (Fig. 6a, b), which may be related to the synergistic effects of Lig and Rg1 on stem cell proliferation and senescence. The area of the CD90 fluorescence signal in the Gel/VZ-CLR group was significantly higher than that in the Gel/VZ-C group, indicating that BMSCs were recruited to the wound site under the action of CXCL12 in both groups but that the action of Lig and Rg1 promoted the enrichment of BMSCs at the wound site. We found that the expression of p-JAK2 and p-STAT3 in groups treated with Lig were significantly upregulated compared to those without treatment (Fig. 6c, d, Additional file 1: Fig. S3). Compared to those in the Gel/VZ-C group, the relative expression levels of p-JAK2 and p-STAT3 in the Gel/VZ-CL group were significantly increased by 3.26- and 1.91-fold, respectively (Fig. 6c, d), indicating that Lig may stimulate the proliferation of the migrated BMSCs by activating the Jak2-Stat3 pathway [46]. Compared to those in the Gel/VZ-C group, the expression levels of p-JAK2 and p-STAT3 in the Gel/VZ-CLR group were significantly increased by 3.73- and 2.09-fold, respectively (Fig. 6c, d), suggesting that Rg1 could enhance the Jak2-Stat3 pathway activation by Lig.

PTEN expression was significantly increased in the groups treated with Lig compared with those without Lig (Fig. 6e, Additional file 1: Fig. S3). The expression levels of PTEN in the Gel/VZ-CLR group were significantly increased by 3.00-fold compared to that in the Gel/VZ-C group (Fig. 6e), which could alleviate cell senescence induced by reactive oxygen species [47, 48]. These results further confirmed that the combination of Lig and Rg1 has a synergistic effect on promoting the proliferation of stem cells by activating the Jak2-Stat3 pathway and alleviating senescence via PTEN.

### ASD promotes angiogenesis and nerve regeneration in vivo

**Fig. 7:**
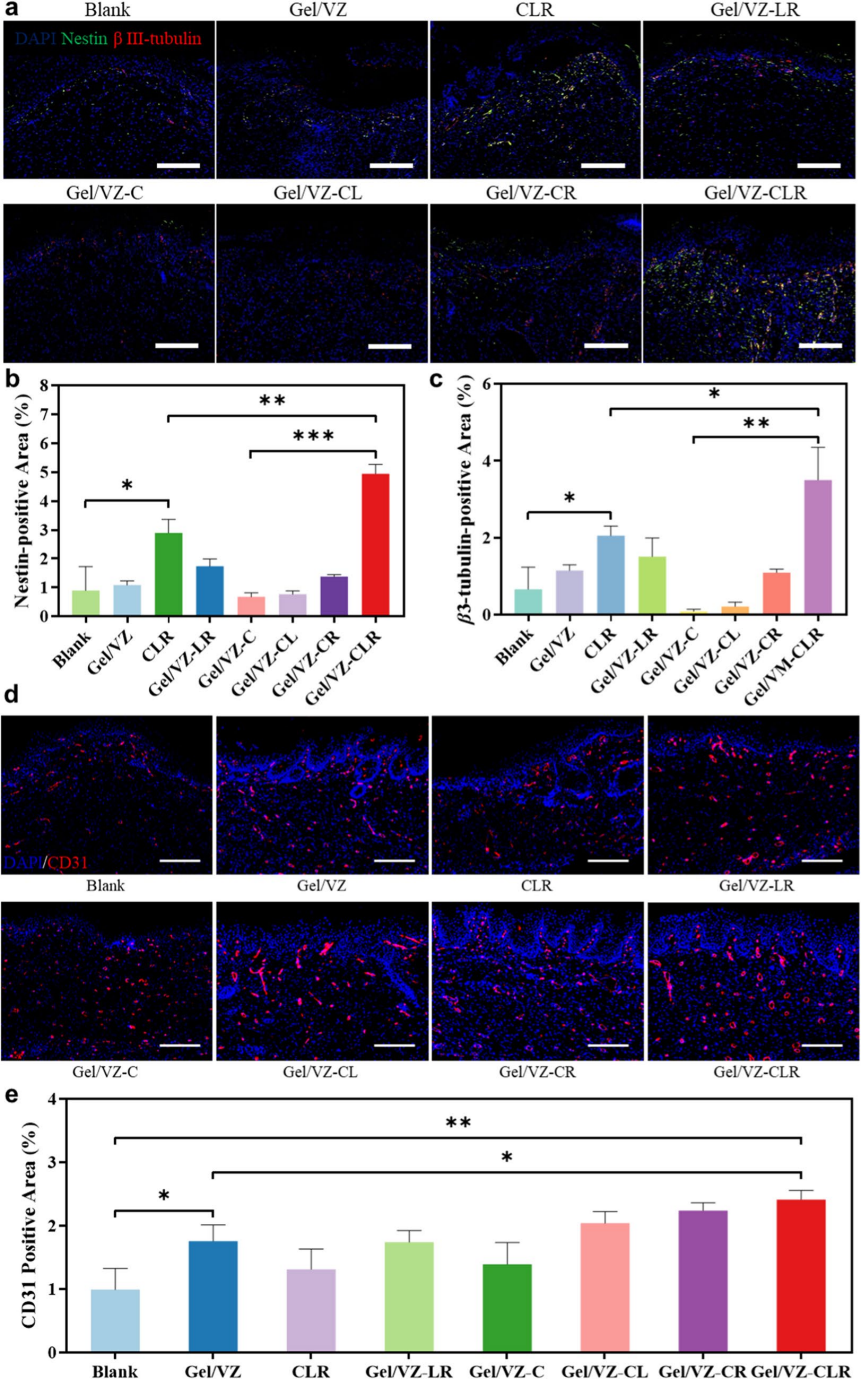
In vivo nerve regeneration and angiogenesis. **a** Immunofluorescence staining and semi-quantitative fluorescence-positive area (Scalebar = 200 μm) of **b** nestin (green) and **c** *β*3-tubulin (red) in healed skin tissue on day 17 post-treatment. **d** Immunofluorescence staining and (Scalebar = 200 μm). **e** semi-quantitative fluorescence-positive area of CD31 (red) in healed skin tissue on day 17 post-treatment. *p < 0.05, **p < 0.01, ***p < 0.001 compared to the Blank group

The above results demonstrated that the Gel/VZ-CLR dressing effectively recruited BMSCs to the wound site within 3 days by simultaneously promoting stem cell proliferation and increasing the expression of anti-senescence proteins, thus showing great potential for stem cell-based tissue regeneration. Therefore, we investigated the neuroregenerative effects induced by the ASD in vivo. We observed nestin and *β*3-tubulin fluorescence signals in the groups containing Rg1 (Fig. 7a). The signals of nestin and *β*3-tubulin in the Gel/VZ, CLR, Gel/VZ-LR, and Gel/VZ-C groups were mostly concentrated to the new epidermis, whereas no obvious signals of nestin or *β*3-tubulin were found in the new dermis. In the healed skin tissue of the Gel/VZ-CLR group, obvious signals of nestin and *β*3-tubulin were observed in the regenerated epidermis and dermis, indicating that Gel/VZ-CLR effectively induced BMSC differentiation in vivo. Compared to the that in the blank group, CLR treatment significantly increased the area of nestin and *β*3-tubulin signals (Fig. 7b, c), confirming that the combination of CXCL12, Lig, and Rg1 promoted the differentiation of BMSCs in vivo. Compared with that in the Gel/VZ group, the Gel/VZ-RL treatment significantly enhanced the nestin fluorescence signal in the healed skin, indicating that Gel/VZ-RL can also promote nerve regeneration with limited recruitment of BMSCs, which may be related to the proliferative and anti-senescence effects of Lig as well as the neuroprotective effect of Rg1.

Compared to those in the Gel/VZ-C group, cells of the Gel/VZ-CLR group showed the highest expression levels of both nestin and *β*3-tubulin, with fluorescent areas increased by 7.4- and 43.8-fold, respectively. This enhancement can be attributed to the continuous promotion of stem cell proliferation and neural differentiation by Lig and Rg1 delivered by the ASD. Interestingly, the significantly enhanced nerve regeneration in the ASD-treated skin can be partially attributed to the pro-angiogenic effect of the ASD. CD31 immunohistological staining indicated that Gel/VZ treatment promoted angiogenesis in healed skin on day 17 post-treatment (Fig. 7d). CD31 expression in the Gel/VZ group was significantly increased by 1.77-fold compared to that in the blank group, while Gel/VZ-CLR treatment further increased CD31 expression by 2.44-fold compared to the blank group (Fig. 7e). The neovessels provide nutrients for nerve regeneration and directional cues for migrating Schwann cells, which guide the regrowth of axons between new tissues [49]. These results indicate that the ASD is an efficient platform that enhances neovascular formation and promotes the enrichment of BMSCs at the wound site while accelerating their differentiation into different neural lineages.

### Evaluation of wound regeneration

**Fig. 8:**
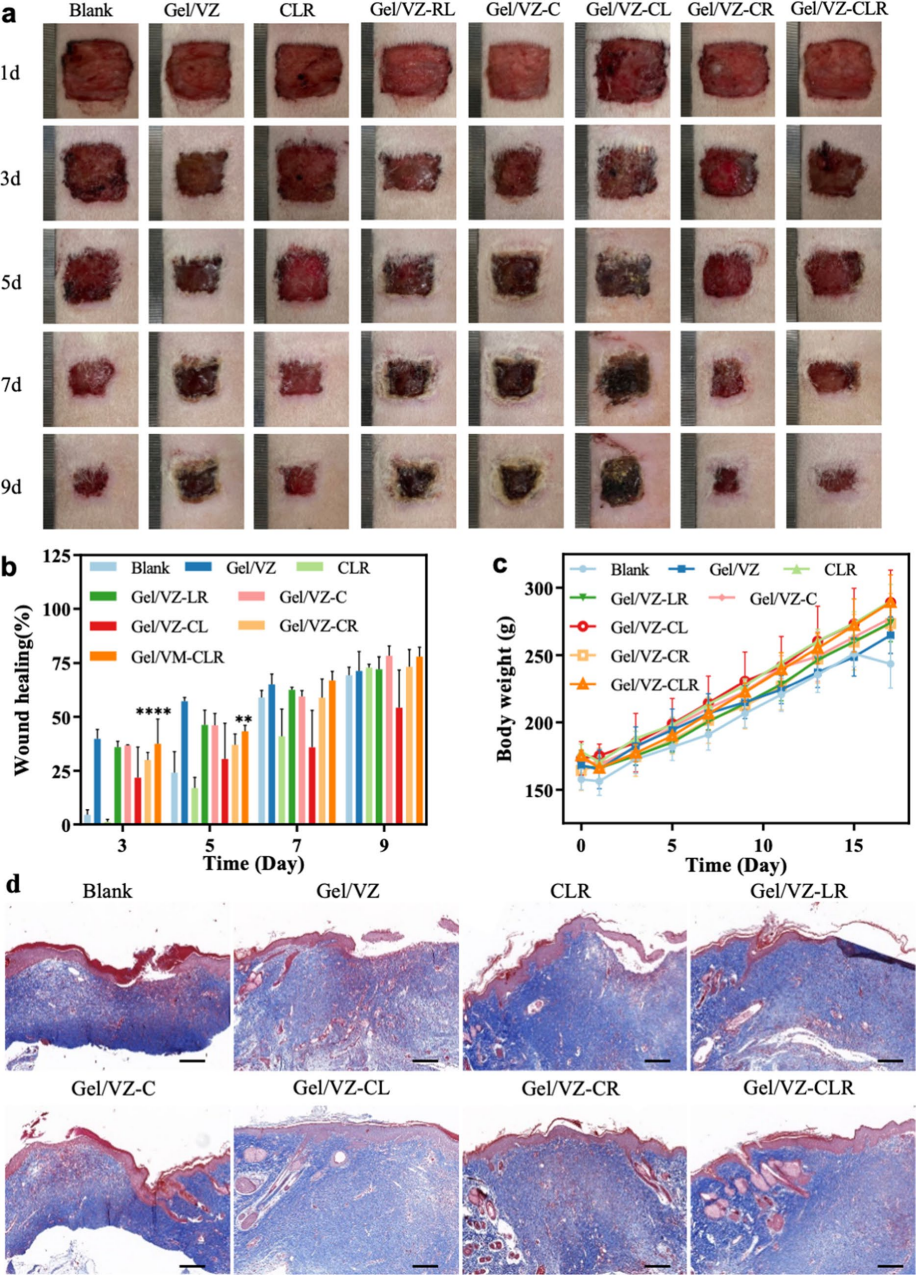
In vivo wound regeneration. **a** Representative images of wounds throughout the healing process. **b** Quantitative analysis of wound healing ratio over time. **c** Body weight of Sprague Dawley (SD) rats during treatment. **d** Masson staining of wound bed in different treatment groups (Scalebar = 200 μm). *p < 0.05, **p < 0.01, ***p < 0.001 compared to the Blank group

Peripheral nerve regeneration plays an important role in the promotion of tissue repair [50, 51]. We further studied the role of Gel/VZ-CLR in promoting wound healing. Scabs were still observed in the Blank, Gel/VZ, and CLR groups 17 d after treatment, while no obvious wounds or scabs were observed in the remaining unmentioned groups (Fig. 8a, Additional file 1: Fig. S4a). Quantitative analysis indicated that the ASD promoted wound healing already in the early stages (Fig. 8b, Additional file 1: Fig. S4b). The Gel/VZ significantly promoted wound healing on days 3 and 5 compared with the blank group. The wound healing effect of the Gel/VZ-CLR group was 8.5- and 1.8-times greater than that of the blank group at 3 and 5 d post-treatment, respectively (Fig. 8b), while maintaining normal body weight (Fig. 8c). The prominent wound healing effect of Gel/VZ-CLR is likely related to the following: (1) The mobility and in situ crosslinking of GelMA hydrogel prepolymers allow for an adequate fit of the wound to establish a relatively isolated and moist environment, which can promote wound healing [52, 53]; (2) Angiogenesis in the early stage of wound repair can promote healing efficiency by promoting material exchange, growth factor transport, and fibroblast migration [54]; and (3) The regenerated nerve in trauma repair may promote wound healing by secreting necessary growth factors [51, 55].

Based on the positive role of the ASD in promoting wound healing, we examined the effect of the ASD on the histomorphology of healed skin using Masson’s staining. The distribution of collagen fibers in the Blank, Gel/VZ, and CLR groups was roughly parallel to that of the new epidermis, indicating that the new tissues did not form the reticular interlaced collagen layer of normal skin, and the formation of skin appendages was not observed (Fig. 8d), which corresponds with the characteristics of scar formation [56, 57]. In comparison, the formation of other skin appendages were observed in the Gel/VZ-RL, Gel/VZ-C, and Gel/VZ-CLR groups; collagen fibers in the Gel/VZ-RL and Gel/VZ-CLR groups were interlaced and resembled those in normal skin [58], indicating that the ASD dressings can promote the functional repair of skin wounds [59]. The thickness of the granulation tissue is another important index for evaluating the degree of wound repair [60]. Compared with that in the blank group, the granulation tissue thickness of the Gel/VZ group increased by 516.6 μm, while that of the Gel/VZ-CLR group increased 1094.8 μm (Additional file 1: Fig. S4). The significantly increased wound healing rate associated with Gel/VZ and Gel/VZ-CLR may also be related to the increased thickness of the granulation tissue [61].

Based on the gradually increasing body weights and lack of pathological changes in the hematoxylin and eosin (H&E) staining of the heart, liver, spleen, lung, and kidney in all treatment groups compared with the blank group (Additional file 1: Fig. S5), we confirmed that the Gel/VZ-CLR smart gel dressing showed good biocompatibility and safety in wound repair.

## Discussion

In this study, we successfully constructed an all-in-one smart dressing platform, the ASD, based on VO_2_@ZIF-67-incorporated GelMA loaded with multiple therapeutic components, which establishes an effective artificial niche for angiogenesis and in situ neural regeneration. The nanocomposite VO_2_@ZIF-67 allows the ASD to release angiogenic Co^2+^ in an on-demand manner based on the pH of the wound site. All wounds treated with the Gel/VZ showed an enhanced CD31 expression compared to that of the PBS treatment on day 17 (Fig. 7d) along with faster wound contraction (Fig. 8b), while the rats maintained normal growth (Fig. 8c) and experienced little to no toxic side effects (Additional file 1: Fig. S5). The ASD simultaneously released CXCL12, Lig, and Rg1 in a sustained manner to stimulate stem cell migration, inhibit senescence, and induce neural differentiation, respectively, thereby establishing a biomimetic niche maintaining the self-renewal and multipotency of stem cells and accelerating nerve regeneration.

Gel/VZ-CLR-treated wounds showed significantly enhanced nestin and *β*3-tubulin expression compared with Gel/VZ-C-treated wounds (Fig. 7a–c), demonstrating that the co-delivery of Lig and Rg1 promoted the neural differentiation of stem cells. Interestingly, the enhanced nerve regeneration effects of the ASD can be partially attributed to the pro-angiogenic effect of the Gel/VZ: The nerve regeneration effects in Gel/VZ-CLR-treated wounds were significantly enhanced compared to those in CLR-treated wounds, and the enhanced CD31 expression in the Gel/VZ group promoted angiogenesis compared to the blank group (Fig. 7d, e). The neovessels act as channels for nutrient transport and stem cell migration, while also directing migrating Schwann cells, which guide the regrowth of axons across the gap between new tissues to reconnect severed nerves [49]. Angiogenesis thus plays a vital role in tissue regeneration and especially nerve regeneration. These results revealed that wounds treated with the Lig-incorporated ASD showed enhanced stem cell enrichment and the addition of Rg1 further promoted this enhancement, which was related to the Jak2-Stat3 and PTEN pathways, resulting in the increased regenerative potential of stem cells. In summary, the ASD promoted wound regeneration by establishing a favorable hydrogel environment that stimulates angiogenesis through the directional cues of the VZ and induces nerve regeneration by multi-drug delivery.

## Conclusions

The proposed all-in-one smart dressing demonstrates the potential of integrating nanomaterials and multiple therapeutics within GelMA dressings to achieve on-demand delivery of multiple active factors, providing a bottom-up approach to stimulating both angiogenesis and neural regeneration. Our study provides a verified proof-of-concept to promote recruitment, angiogenesis, and neuronal regeneration for endogenous mesenchymal stem cell-based regeneration of multiple functional lineages using a smart dressing.

## Materials and methods

### Materials

The following reagents were purchased from various vendors: vanadium pentoxide (V_2_O_5_), oxalic acid dihydrate (C_2_H_2_O_4_⋅2H_2_O), isopropanol, cobalt nitrate hexahydrate (Co (NO_3_)_2_⋅6H_2_O), methanol, dimethyl imidazole (C_4_H_6_N_2_, Hmim), hydrochloric acid (HCl), ethanol (Sinopharm Chemical Reagent Co., Ltd., China), 3,3′,5,5′-tetramethylbenzidine (TMB, Shanghai Aladdin Biotechnology Co., Ltd., China), tryptone (LP0024, OXOID, USA), yeast extract (LP0021, OXOID, USA), agarose (Bacto agar, BD, USA), sodium chloride (NaCl, Guoyao Group Chemicals Co., Ltd., China), low growth factor matrix gel (Corning, USA), Tissue Protein Extraction Reagent (T-PER, Saimofeishier Technology Co., Ltd., China), Halt Protease and Phosphatase Inhibitor Cocktail (100× , Saimofeishier Technology), BCA Protein Concentration Determination Kit (Enhanced, Shanghai Biyuntian Biotechnology Co., Ltd., China), PVDF Transfer Membrane (Millipore, USA), ECL DualVue WB Marker (GE Healthcare, USA), SuperSignal West Dura Extended Duration Substrate (Semerfeld Technology Co., Ltd., China), Rg1 and Lig (Dingrui Chemical Co., Ltd., Shanghai, China), CCK-8 kit (Shanghai Biyuntian Biotechnology), 4% paraformaldehyde (Wuhan Boshide Bioengineering Co., Ltd., China), calcium fluorescent probes (Fluo A4, S1060, Shanghai Beyotime Biotechnology Co., Ltd., China), potassium chloride (KCl, Shanghai Macklin Biochemical Technology Co., Ltd., China), GelMA (GM-60, Suzhou Yongquan Intelligent Manufacturing Co., Ltd., China), Rat SDF-1α (CXCL12, Peprotech, USA), and Rat SDF-1 ELISA Kit (Shanghai Enzyme-linked Biotechnology Co., Ltd., China).

### Synthesis of hollow VO_2_ and VO_2_@ZIF-67

VO_2_ was synthesized according to a hydrothermal method, as follows: 1.5 g V_2_O_5_ and 1.0 g dihydrate acetic acid were weighed in deionized water, continuously stirred at 70 °C for 60 min, and then added with isopropanol for 30 min at room temperature followed by reaction in a poly-tetrachloroethylene-lined reactor (100.0 mL) at 200 °C for 12 h. The black precipitate was centrifuged, collected, washed three times with ethanol, vacuum dried at 60 °C, and the resulting particles were collected to obtain the hollow VO_2_. The metal–organic framework material ZIF-67 was grown in situ on the prepared VO_2_ surface by co-precipitation. Briefly, 50.0 mg of VO_2_, 290.0 mg of cobalt nitrate hexahydrate, and methanol was added in a water bath for 10 min of ultrasound. The mixed solution was added to 16.5 mg/mL 1-methylimidazole methanol solution and stirred continuously for 20 min. After standing for 24 h, the black precipitate was centrifuged, collected, washed three times with ethanol, and dried in a vacuum oven at 60 °C. After drying, the particles were collected to obtain the composite VO_2_@ ZIF-67 (VZ).

### Characterization of VO_2_ and VO_2_@ZIF-67

The morphologies and structures of VO_2_ and VZ were described using scanning electron microscopy (SEM, SU8010, Hitachi, Japan) and transmission electron microscopy (JEM-2100F, JEOL, Japan). The images were processed using ImageJ software to obtain the particle size distributions of VO_2_ and VZ. The nitrogen adsorption and desorption curves of VO_2_ and VZ were obtained using an automatic specific surface area and micropore analyzer. The elemental distribution of the VZ was analyzed by transmission electron microscopy (TEM) with an attachment to the spectrometer.

### Preparation of gel/VZ

We dissolved 50.0 mg Lithium acylphosphinate salt (LAP) in 20.0 mL of ultrapure water to prepare a 2.5 mg/mL LAP aqueous solution. GelMA [33, 62] was dissolved in LAP aqueous solution in a water bath at 37 °C to prepare 10% (w/v) GelMA solution. After GelMA was completely dissolved, VZ was added and vortexed 1 min prior to exposure to 405 nm light to prepare 200 μg/mL Gel/VZ, which was stored in a refrigerator at 4 °C.

### Rheological analysis of Gel/VZ

Rheological measurements of the ASD were performed with a rheometer (Anton Paar) using three different methods: (1) The strain amplitude sweep tests of GelMA and Gel/VZ were conducted at a fixed angular frequency (1 rad/s) with γ = 0.1%–300%; (2) The self-restoring abilities of GelMA and Gel/VZ were investigated by step frequency scanning at 37 °C with a fixed frequency of 1 rad/s. Amplitude oscillatory strains were alternated between little strain (γ = 1%) to greater strain (γ = 150%), each lasting for 60 s; and (3) The Frequency scanning of GelMA and Gel/VZ was carried out at a fixed strain (γ = 1%) in the range of ω = 0.1−100 rad/s.

### pH-responsive Co^2+^ release of ASD

For pH responsive Co^2+^ release, Gel/VZ containing 400 μg/mL VZ was prepared and immersed in 5 mL solution with a pH of 7.4, 6.8, or 5.4 in a rotary shaker at 100 rpm, 37 °C. We supplemented 0.5 mL of the solution with fresh solution (0.5 mL) at predetermined time intervals. The amount of Co^2+^ released was determined using inductively coupled plasma mass spectrometry (NexION 300X, PerkinElmer). For pH responsive angiogenesis, 80 μL growth factor-reduced Matrigel basement membrane matrix (Corning) was added to individual wells of 48-well plates and allowed to polymerize at 37 °C for 30 min. After treatment with Gel/VZ at pH 7.4, 6.8, or 5.4 and PBS for 24 h, HUVECs were seeded onto solidified Matrigel at a density of 2 × 10^4^ cells/well. The enclosed vessel networks were photographed under a microscope after 12 h incubation at 37 °C. The obtained images were analyzed using the Angiogenesis Analyzer in ImageJ for the length of tubules and the number of reticular structures.

### Drug release profiles from ASD

We added 200 ng CXCL12, 2 mg Rg1, and 2 mg Lig to 600 μL Gel/VZ gel containing 200 μg/mL VZ, mixed the solution, and placed it in a square mold under 405 nm light for 1 min to prepare the ASD. The ASD was placed in a dialysis bag (COMW = 8000–14,000 KDa) with 5 mL PBS and then soaked in 15 mL PBS in a rotary shaker at 180 rpm. Then, 1 mL of supernatant was sampled for drug content determination at a predetermined time point, and 1 mL fresh PBS was added after each sampling. CXCL12 concentration was determined using the ELISA kit, and Rg1 and Lig concentrations were determined by HPLC under the conditions described in the Additional file 1.

### Isolation and culture of BMSCs

BMSCs were isolated and cultured as previously described [63]. SD rats were supplied by Shanghai Laboratory Animal Co. (SLAC), Ltd., China. All experimental procedures were performed in accordance with the Zhejiang University guidelines for the welfare of experimental animals. Briefly, rat femurs were excised from the epiphysis and bone marrow was flushed out using a syringe with Dulbecco’s modified Eagle’s medium (DMEM, Gibco BRL) supplemented with 10% fetal bovine serum (FBS, Gibco BRL), l-glutamine, penicillin (50 U/mL), and streptomycin (50 U/mL). The cell suspension was placed in a 25 cm^2^ tissue culture flask (Corning) and cultured at 37 °C in 5% CO_2_. Subconfluent first passage cells were detached from the flask with 0.25% trypsin–EDTA for 2 min at 37 °C. The second- to fifth-generation BMSCs were used in subsequent experiments.

### Cell recruitment assay

BMSC migration was tested using an RTCA DP instrument (ACEA Biosciences Inc.). Firstly, Gel/VZ-C and Gel/VZ-CLR were prepared with a VZ concentration of 200 μg/mL, CXCL12 concentration of 0.83 ng/μL, and Rg1 and Lig concentrations of 0.12 mg/mL. Then, 20 μL of Gel/VZ-C, Gel/VZ-CLR, or PBS was added to the bottom chamber of modified 16-well plates (E-Plate 16, ACEA Biosciences Inc.) and cross-linked by 405 nm light for 1 min. Subsequently, 145 μL of serum-free cell culture medium was added into the bottom chamber, the top chamber of the E-Plate 16 was filled with serum-free medium, and the membrane was hydrated and preincubated in the CO_2_ incubator (HF90, Health Force) at 37 °C for 1 h before obtaining a background measurement. After this incubation period, the BMSC suspension was seeded into the top chamber at 5 × 10^4^ cells in 100 µL. The E-Plate 16 was assembled by placing the top chamber onto the bottom chamber and then placed in the RTCA DP station to automatically monitor the impedance value every 5 min for 48 h, which was expressed as a cell index value. All data were recorded using RTCA software.

### BMSCs proliferation stimulated by multiple drugs

BMSCs were seeded in 96-well plates at a density of 5 × 10^3^ cells/well and incubated in 5% CO_2_. After the cells adhered to the well, the culture medium was replaced, and PBS, Rg1, Lig, Rg1 + Lig, and VZ + Rg1 + Lig were added (the concentrations of Rg1, Lig, and VZ were 15 μg/mL, respectively). After 24 h of culturing in a CO_2_ incubator, the culture medium was removed according to the CCK-8 kit instructions. The culture medium was gently washed twice with sterile PBS; 100 μL complete medium and 10 μL working solution were added to each well. After incubation for 1 h, the absorbance of the supernatant was measured at 450 nm using a microplate reader.

### SA-β-gal staining

Fifth-generation BMSCs were seeded onto 6-well plates at a density of 1 × 10^5^ cells/well. After treatment with PBS, Rg1, Lig, Rg1 + Lig, or VZ + Rg1 + Lig were added (the concentrations of Rg1, Lig, and VZ were 15 μg/mL, respectively) for 24 h. The BMSCs were then stained with SA-β-gal staining solution (Beyotime) and incubated overnight at 37 °C. Images were captured using an inverted microscope (Nikon, Tokyo, Japan), and the number of positive cells was calculated using ImageJ software.

### Neural differentiation of BMSCs in vitro

BMSCs were seeded onto confocal culture dishes at a density of 4 × 10^4^ or 2 × 10^4^ cells/dish, and incubated overnight in the CO_2_ incubator at 37 °C. PBS, Rg1, Lig, Rg1 + Lig, or VZ + Rg1 + Lig were added (the concentrations of Rg1, Lig, and VZ were 15 μg/mL, respectively) every two days. After being cultured for 7 d, the cells were washed with PBS, fixed in 4% paraformaldehyde (Solarbio Life Science) for 30 min, permeabilized with 0.1% Triton X-100 (Sigma-Aldrich) for 20 min, and blocked with 10% goat serum (Boster Biological Technology) for 30 min. The samples were then incubated overnight at 4 °C with anti-nestin antibody (Omnimab) and anti-*β*3-tubulin antibody (Cell Signaling Technology), and detection was achieved by subsequent incubation with FITC-conjugated goat anti-mouse IgG H&L (Beyotime) and CY3-conjugated goat anti-rabbit IgG H&L (Boster Biological Technology) for 1 h at 37 °C, followed by DAPI staining and imaging under a confocal fluorescence microscope (BX61, Olympus).

### Calcium imaging assay

After treatment with PBS or VZ-LR and culturing for 7 d, the changes in Ca^2+^ were measured using the fluorescent Ca^2+^ indicator Fluo-4 AM (Beyotime) by confocal microscopy, as previously described. Briefly, cells were washed twice with Hank’s Balanced Salt Solution (HBSS) twice and loaded with 2 μM Fluo-4 AM for 30 min in the dark. The cells were rinsed with HBSS twice and incubated for another 20 min at 37 °C to ensure that Fluo-4 AM had completely transformed into Fluo-4. Images were captured using a laser scanning confocal microscope (Nikon A1R). Fluo-4 AM was excited at a wavelength of 488 nm. After the fluorescent signal stabilized (F_0_), 4.1 mg/mL KCl was added to excite the cells, and the excitation (F_t_) was recorded in real-time for 3 min with 10-s intervals. The changes in Ca^2+^ were reflected by relative fluorescence calculated as ΔF = F_t_ − F_0_.

### Full-thickness wound model construction

We purchased 56 male SD rats, each weighing 140–160 g, from the SLAC. All animals were maintained under constant conditions (temperature = 25 ± 1 °C), with free access to standard chow and drinking water. All animal experimental procedures were performed in accordance with the guidelines and protocols of the Animal Experimental Ethics Committee of the Zhejiang University. The animals were anesthetized with an intraperitoneal injection of 3% sodium pentobarbital (30 mg/kg). Full-thickness excision wounds were made symmetrically (1.5 × 1.5 cm) by scalpel excision on the depilated back of each rat. Rats were randomly divided into eight experimental groups (n = 7 per group): blank, Gel/VZ (VZ composite with GelMA hydrogel and blank vector), CLR (CXCL12, Lig, and Rg1 mixed), Gel/VZ-LR (VZ composite with GelMA hydrogel loaded with Lig and Rg1), Gel/VZ-C (VZ composite with GelMA hydrogel loaded with CXCL12), Gel/VZ-CL (VZ composite with GelMA hydrogel loaded with CXCL12 and Lig), Gel/VZ-CR (VZ composite with GelMA hydrogel loaded with CXCL12 and Rg1), and Gel/VZ-CLR (VZ composite with GelMA hydrogel loaded with CXCL12, Lig, and Rg1). After treatment, all groups were dressed with transparent tegaderm to prevent infection and wound rehydration. All groups were treated every 2 d post-surgery for a total of six times. Wound healing was recorded by taking a picture every 2 d and measuring the wound area. The weights of the rats were also recorded. The wound-healing rate was calculated by dividing the difference between the area on day 0 and day *n* by the area on day 0.

### Histological analysis

Histological analysis was performed on healed skin tissues and organs 17 days after wound treatment. Samples were fixed in 4% buffered paraformaldehyde, dehydrated, and then embedded in paraffin or OCT compound for slice preparation. The sample sections (5-μm thick) were stained with Masson’s trichrome staining (Keygen Biotech) according to the manufacturer’s protocol. The stained skin sections were observed using a laser scanning confocal microscope (VS120, Olympus). Organs including the heart, liver, spleen, lung, and kidney were extracted and cut into smaller sections, fixed in 4% paraformaldehyde, embedded in paraffin, and sectioned into 5-μm-thick slices. The organ sections were stained with H&E (Keygen Biotech) and visualized using laser scanning confocal microscopy for the histological study of toxicity.

### Immunofluorescent staining

Wound tissues were sampled on days 3 and 17 after treatment, embedded in an optimal cutting temperature compound, frozen, and sliced into 10-µm-thick sections at − 22 °C. Sections were then treated with primary antibodies against rabbit anti-CD31 (Abcam) overnight at 4 °C, followed by a 50 min treatment with goat anti-rabbit IgG secondary antibody (Abcam) at 37 °C and 10 min treatment with 4′,6-diamidino-2-phenylindole (DAPI). The stained slides were observed under an Olympus VS200 fluorescence microscope (Japan). To visualize the migration of BMSCs and regenerated nerves in vivo, tissue sections on day 3 and 17 were stained with antibodies against CD90 (ProteinTech), nestin (ProteinTech), and *β*3-tubulin (Cell Signaling Technology). CD90, nestin, and *β*3-tubulin signals were visualized using FITC- and CY3-conjugated secondary antibodies (Thermo Pierce). Nuclei were stained with DAPI. Using a laser scanning confocal microscope (VS200, Olympus), images of the sections were obtained for three randomly selected areas for the quantification of fluorescence intensity. All images were post-processed and quantified using ImageJ software.

### Western blot analysis

Samples were obtained from the rat wound area and homogenized using T-PER tissue protein extraction reagent (Thermo Fisher Scientific). The protein concentration was determined using a bicinchoninic acid protein assay (Beyotime). Western blot analysis was performed using 10% sodium dodecyl sulfate–polyacrylamide gel electrophoresis. After the proteins were transferred onto PVDF membranes (Millipore), they were probed with antibodies against p-JAK2 (Cell Signaling Technology), JAK2 (Cell Signaling Technology), p-STAT3 (Cell Signaling Technology), STAT3 (Cell Signaling Technology), PTEN (Cell Signaling Technology), and GAPDH(Abcam) by incubation at 4 °C overnight. After washing with T-TBS, the membranes were incubated with the corresponding secondary antibodies (Thermo Pierce) for 1 h at room temperature. The blots were developed using SuperSignal West Dura Extended Duration Substrate (Thermo Pierce) and recorded on X-ray film (Fuji super RX); the bands were quantified using Image J.

### Statistical analysis

All data were analyzed by one-way analysis of variance and expressed as the mean ± standard deviation (SD). Student’s *t*-test was used to evaluate statistical significance, with *p* < 0.05 considered statistically significant.

## Supplementary Information

**Additional file 1: **Additional materials. **Figure S1.** Particle size distribution of VO_2_ and VZ. **Figure S2.** SA-β-gal staining of stem cells treated with different drugs (Scale bar = 200 μm). **Figure S3.** Western blot analysis of p-STAT3, STAT3, P-JAK2, JAK2 and PTEN in the wounds treated with different groups on day 3. **Figure S4.** In vivo wound regeneration. **a** Representative images of wounds throughout the healing process. **b** Quantitative analysis of wound healing ratio over time. **Figure S5.** Quantitative analysis of the granulation tissue thickness in different groups. *p < 0.05, **p < 0.01, ***p < 0.001 versus Blank group. **Figure S6.** H&E staining of major organs collected from SD rats treated with different groups (Scale bar = 50 μm).

**Additional file 2: Movie S1.** Control.

**Additional file 3: Movie S2.** VZ/RL.

## Data Availability

All data needed to evaluate the conclusions in the paper are present in the paper and/or the additional materials. Additional data available from authors upon request.
