# Supplementary material for: All-in-one smart dressing for simultaneous angiogenesis and neural regeneration
Source: J Nanobiotechnology. 2023 Feb 3;21:38. doi: 10.1186/s12951-023-01787-5 (PMC9896818; doi:10.1186/s12951-023-01787-5)
Supplement: Supplementary file 1 — Additional file 1: Additional materials. Figure S1. Particle size distribution of VO2 and VZ. Figure S2. SA-β-gal staining of stem cells treated with different drugs (Scale bar = 200 μm). Figure S3. Western blot analysis of p-STAT3, STAT3, P-JAK2, JAK2 and PTEN in the wounds treated with different groups on day 3. Figure S4. In vivo wound regeneration. a Representative images of wounds throughout the healing process. b Quantitative analysis of wound healing ratio over time. Figure S5. Quantitative analysis of the granulation tissue thickness in different groups. *p < 0.05, **p < 0.01, ***p < 0.001 versus Blank group. Figure S6. H&E staining of major organs collected from SD rats treated with different groups (Scale bar = 50 μm). [file 12951_2023_1787_MOESM1_ESM.docx]

Additional file 1

**All-in-one Smart Dressing for Simultaneous Angiogenesis and Neural Regeneration**

Tiejun Yuan^a^, Minhong Tan^a,c^, Yang Xu^a^, Qiyao Xiao^a^, Hui Wang^c^, Chen Wu^c^, Fulun Li^d^, Lihua Peng^a,b^*

^a^ College of Pharmaceutical Sciences, Zhejiang University, Hangzhou, 310058, PR China

^b^ State Key Laboratory of Quality Research in Chinese Medicine, Macau University of Science and Technology, Macau, PR China

^c^ College of Materials Science and Engineering, State Key Laboratory of Silicon Materials, Zhejiang University, Hangzhou 310027, PR China

^d^ Department of Dermatology, Yueyang Hospital of Integrated Traditional Chinese and Western Medicine, Shanghai University of Traditional Chinese Medicine, Shanghai 200437, PR China

Corresponding author

Li-Hua Peng, Ph.D. Associate Professor.

College of Pharmaceutical Sciences, Zhejiang University, China

Tel/Fax: +86-571-88981231; Email: [lhpeng@zju.edu.cn](mailto:lhpeng@zju.edu.cn)

**Determination of Rg1 by High-Performance Liquid Chromatography**

Phenomenex C_18_ chromatographic column, mobile phase acetonitrile (A) -water (B) gradient elution (0 ~ 5 min, 9 % A ; 5-10 min, 19 % -29 % A ; 1015 min, 29 % A ; 15 – 20 min, 29 % – 40 % A ; 20 – 25 min, 40 % – 19 % A ; 25 ~ 30 min, 19 % A), flow rate 1.0 mL / min, column temperature, detection wavelength 203 nm.

**Determination of Lig by High-Performance Liquid Chromatography**

Phenomenex C_1_8 chromatographic column, mobile phase methanol (A) -0.2 % phosphoric acid aqueous solution (B) gradient elution (0 ~ 5 min, 45 % A; 5 – 15 min, 45 % – 50 % A), flow rate 1.0 mL / min, column greenhouse temperature, detection wavelength 203 nm.

**Figure S1.** Particle size distribution of VO_2_ and VZ.


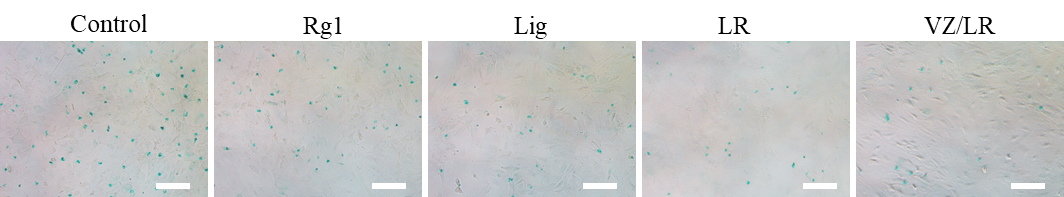


**Figure S2.** SA-β-gal staining of stem cells treated with different drugs (Scale bar = 200 μm).


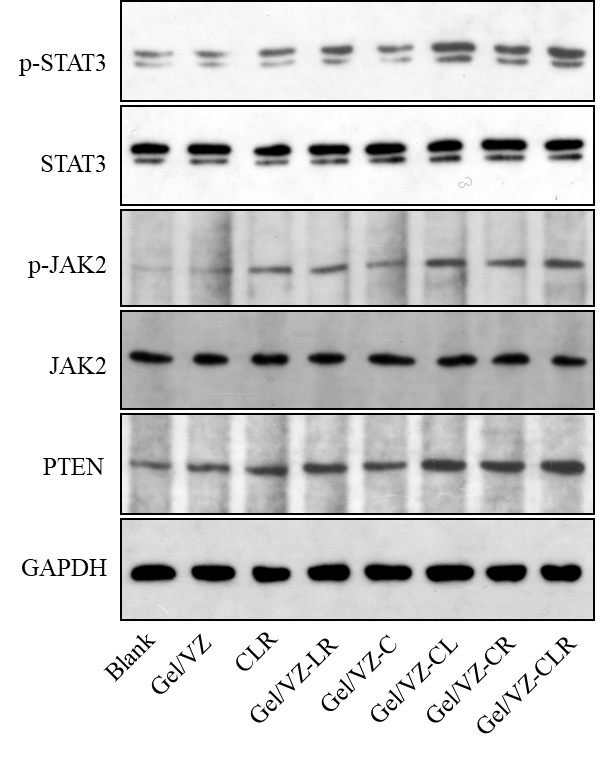


**Figure S3.** Western blot analysis of p-STAT3, STAT3, P-JAK2, JAK2 and PTEN in the wounds treated with different groups on day 3.


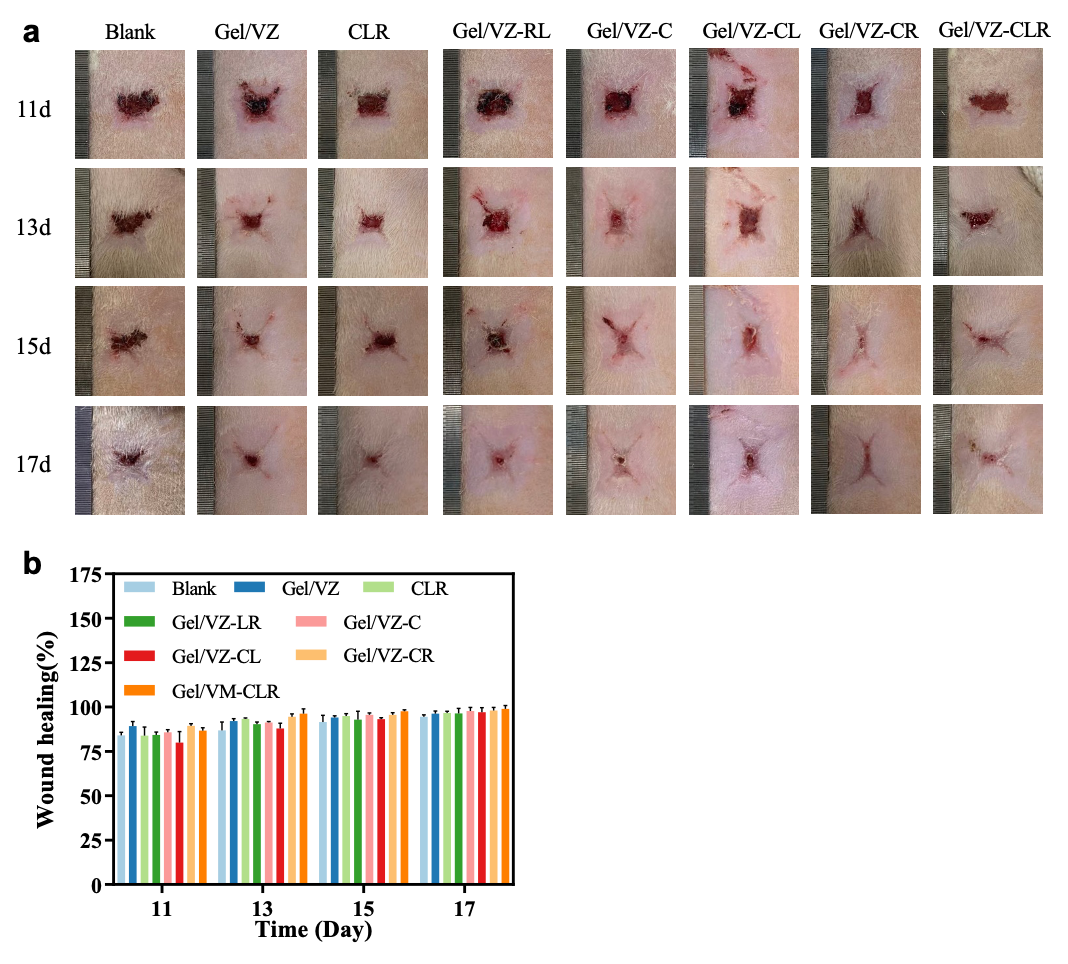


**Figure S4.** *In vivo* wound regeneration. a) Representative images of wounds throughout the healing process. b) Quantitative analysis of wound healing ratio over time.

**Figure S5.** Quantitative analysis of the granulation tissue thickness in different groups. **p*<0.05, ***p*<0.01, ****p*<0.001 versus Blank group.


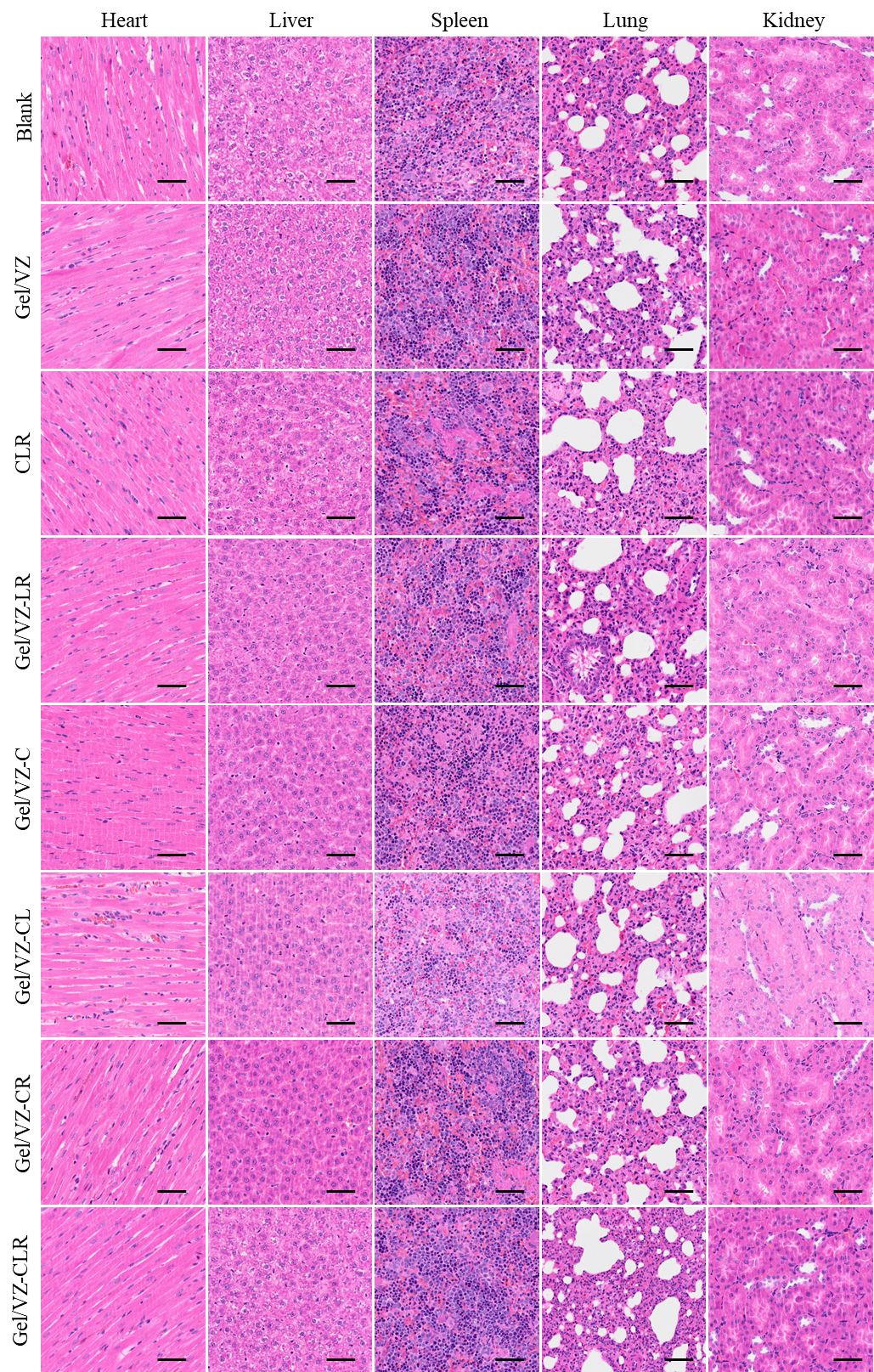


**Figure S6**. H&E staining of major organs collected from SD rats treated with different groups (Scale bar = 50 μm).
